# Supplementary material for: Modeling the relative risk of incidence and mortality of select vaccine-preventable diseases by wealth group and geographic region in Ethiopia
Source: PLOS Glob Public Health. 2022 Aug 31;2(8):e0000819. doi: 10.1371/journal.pgph.0000819 (PMC10004450; doi:10.1371/journal.pgph.0000819)
Supplement: S1 Text — (DOCX) [file pgph.0000819.s001.docx]

**Modeling the relative risk of select vaccine-preventable diseases by wealth group and geographic region in Ethiopia**

Sarah Bolongaita,^1^ Dominick Villano,^1^ Solomon Tessema Memirie,^2^ Mizan Kiros Mirutse,^3^
Alemnesh H. Mirkuzie,^4^ Sophia Comas,^1^ Eva Rumpler,^5^ Stephanie M. Wu,^6^ Ryoko Sato,^1^
Angela Y. Chang,^7^ Stéphane Verguet^1^

^1^ Department of Global Health and Population, Harvard T.H. Chan School of Public Health, Boston, MA, USA

^2^ Addis Center for Ethics and Priority Setting, College of Health Sciences, Addis Ababa University,
Addis Ababa, Ethiopia

^3^ Ministry of Health, Federal Democratic Republic of Ethiopia, Addis Ababa, Ethiopia

^4^ National Data Management Center for Health, Ethiopian Public Health Institute, Addis Ababa, Ethiopia

^5^ Department of Epidemiology, Harvard T.H. Chan School of Public Health, Boston, MA, USA

^6^ Department of Biostatistics, Harvard T.H. Chan School of Public Health, Boston, MA, USA

^7^ Danish Institute for Advanced Study, University of Southern Denmark, Odense, Denmark

Correspondence: Stéphane Verguet, 677 Huntington Avenue, Boston, MA 02115. Email: verguet@hsph.harvard.edu

**Supporting Information**

[1. Additional figures 2](#_Toc109406709)

[2. Detailed methodology 3](#_Toc109406710)

[3. Selection of risk and prognostic factors 6](#_Toc109406711)

[3.1. Diarrhea – Rotavirus vaccine 6](#_Toc109406712)

[3.2. Human papillomavirus (HPV) – HPV vaccine 9](#_Toc109406713)

[3.3. Measles – Measles containing vaccine (MCV) vaccine 10](#_Toc109406714)

[3.4. Pneumonia – Penta-3 (DTP-hepB-Hib) and pneumococcal conjugate vaccine (PCV) 12](#_Toc109406715)

[4. Complete death results 16](#_Toc109406716)

[5. References 21](#_Toc109406717)

# Additional figures

Fig A. Trends in child mortality and access to health care in Ethiopia by residence and wealth quintile. Source: Demographic and Health Surveys [1–4]. ARI = acute respiratory infection.


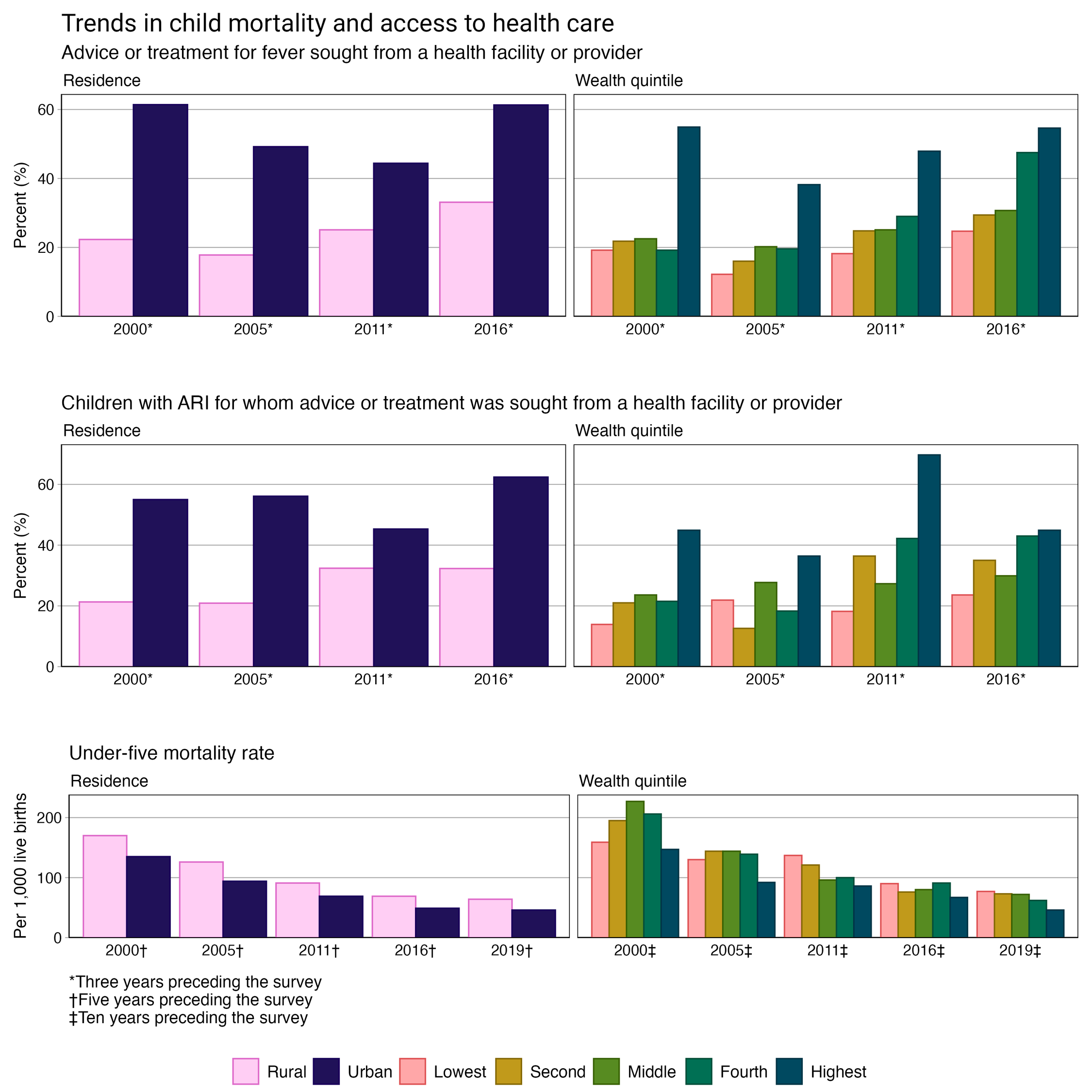


# Detailed methodology

We begin with a population P, a collection of diseases D1, . . . , Dm, and a collection of risk factors RF1, . . . , RFn. To each risk factor RFj we associate a fixed number of possible situations for an individual. For example, there could be three possibilities associated to childhood stunting: not stunted, stunted, and severely stunted, denoted by 1, 2, 3, respectively.

Then, to each disease Di risk factor RFi,j (i denotes disease; j denotes risk factor) and each individual possibility α within RFi,j, there is an associated relative risk RRi,j,α. This relative risk could represent the risk associated to falling ill from disease Di or from dying from disease Di.

Since we consider n risk factors, each individual from population P is assigned an n-dimensional risk factor profile in the form of a list of n numbers. For example, if we consider the risk factors of stunting, underweight, and wasting, the three-dimensional profile (1, 1, 1) would represent an individual who is not stunted, not underweight, and not wasted. The profile (2, 3, 1) would represent an individual who is stunted, severely underweight and not wasted. To each risk factor profile RPk, let PRPk be the number of individuals in population P with risk profile RPk. Similarly, let φi,RPk be a number between 0 and 1 which represents the probability that an individual in PRPk gets sick or dies from disease i.

Since we want to compare the multidimensional profiles to relative risks that each apply to only one risk factor, we introduce the following notation: RP[xj = α] represents all the risk profiles whose j^th^ entry is α. So, from the example in the previous paragraph, RP[x1 = 3] would describe all possible 3-dimensional risk profiles of severely stunted individuals.

Finally, to each disease Di and risk factor RFj we associate the quantity βi,j, which represents the probability that an individual unaffected by risk factor RFj gets sick or dies from disease Di.

With all quantities defined as such, the definition of relative risk then furnishes *m* systems of equations, all linear in $\phi_{i,RP_{k}}$, one for each disease i ($i\in\left[ 1;m \right]$):

| $\left\{ \sum_{RP_{k}\in RP\left[ x_{j}=\alpha\right]} P_{RP_{k}}\phi_{1,RP_{k}}=\beta_{1,j}RR_{1,j,\alpha}\sum_{RP_{k}\in RP\left[ x_{j}=\alpha\right]} P_{RP_{k}} \right\} ,$  … ,  $\left\{ \sum_{RP_{k}\in RP\left[ x_{j}=\alpha\right]} P_{RP_{k}}\phi_{m,RP_{k}}=\beta_{m,j}RR_{m,j,\alpha}\sum_{RP_{k}\in RP\left[ x_{j}=\alpha\right]} P_{RP_{k}} \right\} .$ | (1) |
| --- | --- |

For brevity, we will refer to each of these systems as Aiφ = bi for i = 1, ..., m. The goal is to solve for φ. In general, no solution will exist (as there are more unknowns than equations), so we extend the task to minimizing ∥Aiφ = bi∥_2. We constrain the minimization to the region:

| 1. ≤ φi,RPki ≤ 1; φi,RPki ≤ φi,RPkj | (2) |
| --- | --- |

where each entry of RPki is less than or equal to each entry of RPkj if i<j. Intuitively, this guarantees that φi,RPki corresponds to a probability and that moving to a higher risk profile does not in fact decrease risk. These are the admissible incidence combinations from the Methods section (main text). Although this will almost certainly not yield a unique minimizer, it can deliver a meaningful and useful estimate for possible values of φ for each disease.

The estimates from the first section give rise to a probability distribution associated to the population P. Each individual in P has a multidimensional risk profile RPkj which is then assigned the probability φi,RPki . This yields a probability distribution given by N independent events, where N is the size of P and each event has probability φi,RPki. Since each individual is also assigned a wealth quintile and region, the larger distributions give rise to distributions for each of these subpopulations. This in turn allows us to estimate the distribution of cases and deaths across regions and quintiles. Although perhaps the simplest presentation is in terms of expected value, other statistics are available.

One advantage of comparing ratios of expected values across quintiles and regions is that they are invariant under scaling of the solution vectors of the above systems. Translated to notation, these ratios are independent of βi,j as defined above. This might be considered advantageous since these parameters are equivalent to burden of disease totals, which can be difficult to estimate.

# Selection of risk and prognostic factors

## Diarrhea – Rotavirus vaccine

Table A lists the relevant risk and prognostic factors for morbidity and mortality, respectively, reviewed for diarrhea and rotavirus diarrhea, along with their relative risk magnitudes. Risk factors highlighted in grey were not included for various reasons described beneath the table.

Table A. Relevant risk and prognostic factors for morbidity and mortality, respectively, for diarrhea and rotavirus diarrhea, along with their relative risk magnitudes and corresponding sources.

| Morbidity risk factor | Relative risk (95% CI) | Source ^a^ |
| --- | --- | --- |
| Stunting | z-score < -3 SD: 1.9: (1.3-2.7)  -2 SD < z-score < -3 SD: 1.2 (1.1-1.5) | [5–9] |
| Underweight | z-score < -3 SD: 2.3 (2.1-2.8)  -2 SD < z-score < -3 SD: 1.2 (1.2-1.3) | [5,6,8,9] |
| Unsafe sanitation^b^ | 3.2 (2.9-3.5) | [6–8,10] |
| Wasting | z-score < -3 SD 105.8 (42.2-157.8)  -2 SD < z-score < -3 SD: 23.3 (9.0-35.8) | [5,6,8,9] |
| Having more than 2 children aged under 5 years^c^ | 1.7 | [10] |
| Maternal literacy^c^ | 1.7 | [7,10] |
| Mother’s handwashing not practiced at critical time^c^ | 2.2 | [7] |
| No handwashing with soap^d^ | 1.5 (1.4-1.6) | [6–8] |
| Non-exclusive breastfeeding^d^ | None: 2.2 (1.5-3.2)  Partial: 1.5 (1.0-2.3)  Predominant: 1.2 (1.0-1.7)  Discontinued: 1.3 (1.1-1.5) | [5,6,8,9] |
| Unsafe water source^e^ | 11.1 (4.3-22.9) | [6–8,10] |
| Vitamin A deficiency^c^ | 1.1 (1.0-1.3) | [5,6,8,9] |
| Zinc deficiency^c^ | 1.1 (1.1-1.2) | [5,6,8,9] |
| **Mortality prognostic factor** | **Relative risk (95% CI)** | **Source ^a^** |
| Stunting | z-score < -3 SD: 1.9 (1.3-2.7)  -2 SD < z-score < -3 SD: 1.2 (1.1-1.5) | [5–9] |
| Underweight | z-score < -3 SD: 2.3 (2.1-2.8)  -2 SD < z-score < -3 SD: 1.2 (1.2-1.3) | [5,6,8,9] |
| Wasting | 1.5 (1.4-1.6) | [5,6,8,9] |
| No handwashing with soap^d^ | z-score < -3 SD: 2.3 (2.1-2.8) | [6–8] |
| Non-exclusive breastfeeding^d^ | None: 9.7 (2.4-28.1)  Partial: 3.9 (1.5-8.3)  Predominant: 2.1 1.0-4.6)  Discontinued: 1.3 (1.1-1.5) | [5,6,8,9] |
| Vitamin A deficiency^d^ | 1.1 (1.0-1.3) | [5,6,8,9] |
| Zinc deficiency^d^ | 1.1 (1.1-1.2) | [5,6,8,9] |
| Table is adapted from Chang et al., 2018.  *CI* confidence interval, *SD* standard deviation.  ^a^ Risk and prognostic factors for measles, rotavirus diarrhea, and pneumonia were adapted from Chang et al., 2018.  ^b^ Unsafe sanitation was defined as a household not having access to a flush toilet (piped to sewer system, septic tank, or pit latrine), pit toilet latrine (ventilated or with slab), or composting toilet.  ^c^ Risk factor not included in the analysis due to Demographic and Health Survey (DHS) data unavailability.  ^d^ Risk factor not included in the analysis due to lower relative risk.  ^d^ Risk factor not included in the analysis due to poor data quality and/or poor variable definition.  ^e^ Risk factor not included in the analysis due to computational limitations (using more than four risk factors for joint distribution analysis was considered computationally unfeasible). | | |

### Model inputs

Table B lists quintile- and region-specific vaccination coverage and treatment-seeking behavior proportions, as well as vaccination and treatment efficacies used in the model. Data on vaccination coverage come from the 2016 Ethiopia DHS and refer to the proportion of children who received the second rotavirus vaccination. Data on treatment-seeking behavior come from the 2016 Ethiopian DHS and refer to the proportion of children with diarrhea for whom treatment was sought from a health facility. Rotavirus vaccine efficacy was taken from Li et al., 2021, with an efficacy of 0.50 (0.11-0.72) against rotavirus diarrhea among children under 5 years of age.[11] Diarrhea treatment efficacy was taken from Munos et al., 2010.[12]

Table B. Vaccination coverage and treatment-seeking behavior proportions and corresponding vaccine and treatment efficacies used as model inputs for rotavirus diarrhea.

|  | Vaccination coverage | Treatment-seeking  behavior |  |
| --- | --- | --- | --- |
| **Quintile** |  |  |  |
| Poorest | 0.436 | 0.401 |  |
| Poorer | 0.507 | 0.396 |  |
| Middle | 0.530 | 0.435 |  |
| Richer | 0.646 | 0.440 |  |
| Richest | 0.786 | 0.606 |  |
| **Region** |  |  |  |
| Addis Ababa | 0.917 | NA; 0.625 (fever) |  |
| Afar | 0.233 | 0.530 |  |
| Amhara | 0.591 | 0.400 |  |
| Benishangul-Gumuz | 0.766 | 0.613 |  |
| Dire Dawa | 0.853 | 0.682 |  |
| Gambela | 0.605 | 0.587 |  |
| Harari | 0.613 | 0.545 |  |
| Oromiya | 0.502 | 0.419 |  |
| SNNPR | 0.547 | 0.478 |  |
| Somali | 0.413 | 0.447 |  |
| Tigray | 0.798 | 0.507 |  |
| **Efficacy (95% CI)** | 0.50 (0.11-0.72) | 0.93 (0.83-0.98) |  |
| *CI* confidence interval, *NA* not available (this figure was based on fewer than 25 unweighted cases and was suppressed by the DHS). | | | |

## Human papillomavirus (HPV) – HPV vaccine

Risk factors for HPV infection are primarily related to sexual and reproductive health, including younger age at first sexual intercourse, multiple sexual partners, parity, and use of contraception (Table C).[13–16] Risk factors directly related to sexual activity (i.e. age at first sexual intercourse and lifetime sexual partners) have shown consistent associations with HPV in the literature, including in the Ethiopian context, and data on these indicators is available in the Ethiopian DHS. Parity and limited use of contraceptives have less clear biological pathways linking these behaviors to HPV infection, and there is reason to believe confounding by sexual activity may be present, therefore these risk factors are not included in the analysis.

Individuals with weakened immune systems (including those with HIV/AIDS) may be more likely to contract HPV and are at greater risk for developing health problems if infected.[17] Large observational studies have shown a strong and consistent relationship between HPV-HIV coinfection and cervical intraepithelial neoplasia (CIN) (i.e. pre-cancerous lesions), however the direct relationship between HPV-HIV co-infection and cervical cancer remains controversial, particularly in the African context.[18,19]

Table C. List of the relevant risk factors for infection and prognosis factor for cervical cancer for HPV, along with the extent of the relative risks and the corresponding sources.

| Morbidity risk factor | Relative risk (95% CI) | Source |
| --- | --- | --- |
| Age at first sexual intercourse | <15 years old: OR = 3.32 (2.44-4.53) | [13] |
| Number of lifetime sexual partners | >2: OR = 1.72 (1.26–2.36) | [13–15] |
| HIV co-infection ^a^ | AOR = 4.52 (1.50-13.60) | [20] |
| **Mortality prognostic factor** | **Relative risk (95% CI)** | **Source** |
| HIV co-infection ^a^ | NA | [18,19] |
| *CI* confidence interval, *SD* standard deviation  ^a^ Risk factor not included in the analysis due to Demographic and Health Survey (DHS) data unavailability. | | |

### Model inputs

Table D lists quintile- and region-specific vaccination coverage and treatment-seeking behavior proportions, as well as vaccination and treatment efficacies used in the model. Data on vaccination coverage come from the 2016 Ethiopia DHS and refer to the proportion of children who received the third DPT-HepB-Hib vaccination (the proxy we used for HPV vaccination coverage). The model did not include an analysis of cervical cancer mortality, and therefore seeking treatment was not included as an input for HPV. HPV vaccine efficacy was assumed to be 1 (i.e., 100% effective).

Table D. Vaccination coverage proportion and corresponding vaccine efficacy used as model inputs for HPV.

|  | Vaccination coverage |
| --- | --- |
| **Quintile** |  |
| Poorest | 0.530 |
| Poorer | 0.606 |
| Middle | 0.611 |
| Richer | 0.770 |
| Richest | 0.832 |
| **Region** |  |
| Addis Ababa | 0.957 |
| Afar | 0.201 |
| Amhara | 0.638 |
| Benishangul-Gumuz | 0.762 |
| Dire Dawa | 0.849 |
| Gambela | 0.548 |
| Harari | 0.587 |
| Oromiya | 0.399 |
| SNNPR | 0.590 |
| Somali | 0.363 |
| Tigray | 0.814 |
| **Efficacy** | 1 |

## Measles – Measles containing vaccine (MCV) vaccine

Table E lists the relevant risk and prognostic factors for morbidity and mortality, respectively, reviewed for measles, along with their relative risk magnitudes. Risk factors highlighted in grey were not included for various reasons described beneath the table.

Table E. Relevant risk and prognostic factors for morbidity and mortality, respectively, for measles, along with their relative risk magnitudes and corresponding sources.

| Morbidity risk factor | Relative risk (95% CI) | Source ^a^ |
| --- | --- | --- |
| Stunting | z-score < -3 SD: 2.5 (1.1-6.5)  -2 SD < z-score < -3 SD: 1.5 (1.0-3.2) | [5,6,8] |
| Underweight | z-score < -3 SD: 5.7 (1.8-12.4)  -2 SD < z-score < -3 SD: 2.5 (1.3-5.1) | [5,6,8] |
| Vitamin A deficiency^b^ | 1.4 (1.0-1.9) | [5,6,8,21] |
| Wasting | z-score < -3 SD: 37.9 (5.1-199.1)  -2 SD < z-score < -3 SD: 8.5 (1.3-42.8) | [5,6,8] |
| Having no other children vaccinated at home^c^ | 3.0 | [21,22] |
| Maternal education^c^ | 3.2 | [22] |
| **Mortality prognostic factor** | **Relative risk (95% CI)** | **Source** ^a^ |
| Stunting | z-score < -3 SD: 2.5 (1.1-6.5)  -2 SD < z-score < -3 SD: 1.5 (1.0-3.2) | [5,6,8] |
| Underweight | z-score < -3 SD: 5.7 (1.8-12.4)  -2 SD < z-score < -3 SD: 2.5 (1.3-5.1) | [5,6] |
| Vitamin A deficiency^b^ | 1.4 (1.0-1.9) | [5,6,8,21] |
| Wasting | z-score < -3 SD: 37.9 (5.1-199.1)  -2 SD < z-score < -3 SD: 8.5 (1.3-42.8) | [5,6,8] |
| Age at infection^c,d^ | NA | [21] |
| Infection with complication^c,d^ | NA | [21] |
| Intensity of exposure and patterns of disease transmission^c,d^ | NA | [21] |
| More than one child^c^ | 1.8 | [21,22] |
| Overcrowding^d^ | NA | [21,22] |
| Secondary (versus primary) exposure^c,d^ | NA | [21] |
| *CI* confidence interval, *SD* standard deviation, *NA* not applicable  ^a^ Risk and prognostic factors for measles, rotavirus diarrhea, and pneumonia were adapted from Chang et al., 2018.  ^b^ Vitamin A deficiency was defined as a child 6-59 months (about 5 years) of age who did not receive vitamin A supplements in the six months preceding the DHS interview.  ^c^ Risk factor not included in the analysis due to Demographic and Health Survey (DHS) data unavailability.  ^d^ Risk factor not included in the analysis due to poor data quality and/or poor variable definition. | | |

### Model inputs

Table F lists quintile- and region-specific vaccination coverage and treatment-seeking behavior proportions, as well as vaccination and treatment efficacies used in the model. Data on vaccination coverage come from the 2016 Ethiopia DHS and refer to the proportion of children who received the first measles vaccination. Data on treatment-seeking behavior come from the 2016 Ethiopian DHS and refer to the average proportion of children with diarrhea or ARI for whom treatment was sought from a health facility. MCV vaccine efficacy and measles treatment efficacy were taken from a systematic review by Sudfeld et al., 2010, which estimated that the vaccine had an efficacy of 0.85 (0.83-0.87) against measles disease and that treatment has an efficacy of 0.62 (0.52-0.82).[23]

Table F. Vaccination coverage and treatment-seeking behavior proportions and corresponding vaccine and treatment efficacies used as model inputs for measles.

|  | Vaccination  coverage | Treatment-seeking  behavior |
| --- | --- | --- |
| **Quintile** |  |  |
| Lowest | 0.432 | 0.320 |
| Second | 0.499 | 0.350 |
| Middle | 0.544 | 0.383 |
| Fourth | 0.586 | 0.432 |
| Highest | 0.743 | 0.556 |
| **Region** |  |  |
| Addis Ababa | 0.931 | NA; 0.625 (fever) |
| Afar | 0.301 | 0.472 |
| Amhara | 0.619 | 0.357 |
| Benishangul-Gumuz | 0.708 | 0.515 |
| Dire Dawa | 0.869 | 0.597 |
| Gambela | 0.621 | 0.519 |
| Harari | 0.536 | 0.542 |
| Oromiya | 0.432 | 0.385 |
| SNNPR | 0.576 | 0.423 |
| Somali | 0.481 | 0.358 |
| Tigray | 0.801 | 0.424 |
| **Efficacy (95% CI)** | 0.85 (0.83-0.87) | 0.62 (0.52-0.82) |
| *NA* not available (this figure was based on fewer than 25 unweighted cases and was suppressed by the DHS; treatment seeking for fever alone was used instead) | | |

## Pneumonia – Penta-3 (DTP-hepB-Hib) and pneumococcal conjugate vaccine (PCV)

Table G lists all the relevant risk factors for pneumonia, along with their relative risk magnitudes. The risk factors highlighted in grey were not included for multiple reasons described underneath the table.

Table G. List of the relevant risk factors for pneumonia, along with the extent of the relative risks and the corresponding sources.

| Morbidity risk factor | Relative risk (95% CI) | Source^a^ |  |
| --- | --- | --- | --- |
| Underweight | z-score < -3 SD: 2.6 (1.9-4.4)  -2 SD < z-score < -3 SD: 1.4 (1.2-1.8) | [5,6,8,9] |  |
| Vitamin A deficiency^b^ | 1.6 (1.2-2.0) | [5,6,9,24] |  |
| Wasting | z-score < -3 SD: 47.7 (15.9-94.9)  -2 SD < z-score < -3 SD: 20.5 (7.1-37.9) | [5,6,8,9] |  |
| Crowding (more than 5 people per household)^c^ | 1.4 | [24,25] |  |
| Exposed to household air pollution^d^ | 1.4 | [6,24,25] |  |
| Low birth weight (< 2500g)^d^ | 1.4 | [5,24,25] |  |
| Non-exclusive breastfeeding^d^ | None: 4.5 (95% CI 1.0-18.3)  Partial: 5.4 (95% CI 1.0-20.9)  Predominant: 1.8 (95% CI 1.4-2.3) | [5,6,9,24] |  |
| Parental literacy level^d,e^ | NA | [24,25] |  |
| Secondhand smoke^d^ | 1.2 | [6,24,25] |  |
| Stunting^e^ | z-score < -3 SD: 2.4 (1.1-5.1)  -2 SD < z-score < -3 SD: 1.3 (1.0-2.2) | [5,6,8] |  |
| Zinc deficiency^d^ | 1.8 | [5,6,9,24] |  |
| **Mortality prognostic factor** | **Relative risk (95% CI)** | **Source^a^** |  |
| Stunting | z-score < -3 SD: 2.4 (1.1-5.1)  -2 SD < z-score < -3 SD: 1.3 (1.0-2.2) | [5,6,8,9] |  |
| Underweight | z-score < -3 SD: 2.6 (1.9-4.4)  -2 SD < z-score < -3 SD: 1.4 (1.2-1.8) | [5,6,8,9] |  |
| Vitamin A deficiency^b^ | 1.6 (1.2-2.0) | [5,6,9,24] |  |
| Wasting | z-score < -3 SD: 47.7 (15.9-94.9)  -2 SD < z-score < -3 SD: 20.5 (7.1-37.9) | [5,6,8,9] |  |
| Low birth weight (< 2500g)^d^ | 1.4 | [5,24,25] |  |
| Non-exclusive breastfeeding^d^ | None: 51.4 (95% CI 2.1-325.9)  Partial: 2.8 (95% CI 1.3-5.2)  Predominant: 1.9 (95% CI 1.0-4.1) | [5,6,9,24] |  |
| Secondhand smoke^d^ | 1.2 | [6,24,25] |  |
| Zinc deficiency^d^ | 1.7 | [5,6,9,24] |  |
| *CI* confidence interval, *SD* standard deviation  ^a^ Risk and prognostic factors for measles, rotavirus diarrhea, and pneumonia were adapted from Chang et al., 2018.  ^b^ Vitamin A deficiency was defined as a child 6-59 months (about 5 years) of age who did not receive vitamin A supplements in the six months preceding the DHS interview.  ^c^ Risk factor not included in the analysis due to poor data quality and/or poor variable definition.  ^d^ Risk factor not included in the analysis due to Demographic and Health Survey (DHS) data unavailability.  ^e^ Risk factor not included in the analysis due to lower relative risk. | | | |

### Model inputs

Table H lists quintile- and region-specific vaccination coverage and treatment-seeking behavior proportions, as well as vaccination and treatment efficacies used in the model. Data on vaccination coverage come from the 2016 Ethiopia DHS and refer to the proportion of children who received the third ﻿pneumococcal vaccination (for pneumococcal pneumonia) and the third DPT-HepB-Hib vaccine (for Hib pneumonia). Data on treatment-seeking behavior come from the 2016 Ethiopian DHS and refer to the proportion of children with acute respiratory infection (ARI) for whom treatment was sought from a health facility. If treatment-seeking behavior for ARI was unavailable, treatment seeking for fever was used. Pneumococcal conjugate vaccine efficacy was taken from a systematic review by Lucero et al., 2009, which estimated that the vaccine had an efficacy of 0.06 (0.03–0.09) against clinically diagnosed pneumonia.[26] Hib vaccine efficacy was taken from Griffiths et al., 2012, which estimated that the vaccine had an efficacy of 0.93 (0.83-0.97) against Hib disease.[27] Pneumonia treatment efficacy was taken from Theodoratou et al., 2010, which estimated pneumonia treatment efficacy was 0.70 (0.52-0.82).[28]

Table H. Vaccination coverage and treatment-seeking behavior proportions and corresponding vaccine and treatment efficacies used as model inputs for pneumonia.

|  | Vaccination coverage | | Treatment-seeking  behavior |
| --- | --- | --- | --- |
|  | Pneumococcal | Hib |  |
| **Wealth quintile** |  |  |  |
| Lowest | 0.360 | 0.530 | 0.250 |
| Second | 0.489 | 0.606 | 0.269 |
| Middle | 0.442 | 0.611 | 0.289 |
| Fourth | 0.560 | 0.770 | 0.410 |
| Highest | 0.713 | 0.832 | 0.402 |
| **Region** |  |  |  |
| Addis Ababa | 0.914 | 0.957 | NA; 0.625 (fever) |
| Afar | 0.175 | 0.201 | 0.443 |
| Amhara | 0.605 | 0.638 | 0.291 |
| Benishangul-Gumuz | 0.710 | 0.762 | NA; 0.416 (fever) |
| Dire Dawa | 0.753 | 0.849 | NA; 0.512 (fever) |
| Gambela | 0.461 | 0.548 | NA; 0.450 (fever) |
| Harari | 0.586 | 0.587 | NA; 0.538 (fever) |
| Oromiya | 0.383 | 0.399 | 0.264 |
| SNNPR | 0.486 | 0.590 | 0.432 |
| Somali | 0.349 | 0.363 | 0.322 |
| Tigray | 0.777 | 0.814 | 0.336 |
| **Efficacy** | 0.58 (0.29-0.75) | 0.93 (0.83-0.97) | 0.70 (0.52-0.82) |
| *CI* confidence interval, *NA* not available (this figure was based on fewer than 25 unweighted cases and was suppressed by the DHS; treatment seeking for fever was used instead). | | | |

# Complete death results

The results presented in the main text are an average of methods 1 and 3 in the tables below.

Table I. Complete model results for deaths using three methods: (1) deaths calculated independently from cases, (2) deaths calculated with prognostic factors like risk factors and with treatment-seeking behavior applied, and (3) deaths calculated by applying under-5 mortality rates to cases.

| (a) Rotavirus diarrhea | |  |  |  |  |  |  |  |  |
| --- | --- | --- | --- | --- | --- | --- | --- | --- | --- |
|  | 1. Deaths calculated independently from cases | | | 2. Deaths calculated with prognostic factors like risk factors and with treatment-seeking behavior applied | | | 3. Deaths calculated by applying under-5 mortality rates to cases | | |
|  | “Counter-factual” | Baseline | Maximized vaccination coverage | “Counter-factual” | Baseline | Maximized vaccination coverage | “Counter-factual” | Baseline | Maximized vaccination coverage |
| **Wealth quintile** |  |  |  |  |  |  |  |  |  |
| Poorest | 1.64 (1.54-1.76) | 3.09 (2.51-3.95) | 2.35 (2.16-2.56) | 1.78 (1.66-1.94) | 3.38 (2.77-4.33) | 2.56 (2.33-2.83) | 2.74 (2.53-2.97) | 3.60 (2.96-4.60) | 2.74 (2.53-2.97) |
| Poorer | 1.43 (1.37-1.52) | 2.59 (2.19-3.19) | 2.07 (1.93-2.23) | 1.55 (1.47-1.66) | 2.81 (2.38-3.49) | 2.24 (2.05-2.45) | 2.40 (2.27-2.57) | 3.00 (2.55-3.67) | 2.40 (2.27-2.57) |
| Middle | 1.27 (1.24-1.31) | 2.13 (1.83-2.59) | 1.73 (1.64-1.81) | 1.33 (1.29-1.39) | 2.24 (1.92-2.72) | 1.82 (1.69-1.93) | 1.85 (1.79-1.92) | 2.27 (1.97-2.75) | 1.85 (1.79-1.92) |
| Richer | 1.01 (1.00-1.04) | 1.55 (1.40-1.76) | 1.37 (1.30-1.44) | 1.11 (1.08-1.15) | 1.69 (1.53-1.92) | 1.50 (1.41-1.57) | 1.34 (1.30-1.38) | 1.51 (1.39-1.70) | 1.34 (1.30-1.38) |
| Richest | 1.00 (1.00-1.01) | 1.00 (1.00-1.00) | 1.00 (1.00-1.00) | 1.00 (1.00-1.00) | 1.00 (1.00-1.00) | 1.00 (1.00-1.00) | 1.00 (NA-NA) | 1.00 (1.00-1.00) | 1.00 (1.00-1.00) |
| **Region** |  |  |  |  |  |  |  |  |  |
| Addis Ababa | 1.00 (1.00-1.00) | 1.00 (1.00-1.00) | 1.00 (1.00-1.00) | 1.00 (1.00-1.00) | 1.00 (1.00-1.00) | 1.00 (1.00-1.00) | 1.00 (1.00-1.00) | 1.00 (1.00-1.00) | 1.00 (1.00-1.00) |
| Afar | 3.63 (3.30-4.10) | 7.56 (5.14-11.62) | 4.40 (3.98-4.96) | 3.80 (3.42-4.30) | 7.98 (5.53-12.53) | 4.61 (4.12-5.21) | 5.39 (4.88-6.09) | 9.24 (6.35-14.74) | 5.39 (4.88-6.09) |
| Amhara | 2.70 (2.46-3.02) | 5.46 (4.25-7.43) | 4.07 (3.64-4.56) | 2.91 (2.64-3.27) | 5.89 (4.63-8.17) | 4.37 (3.87-5.00) | 3.88 (3.52-4.34) | 5.20 (4.12-7.10) | 3.88 (3.52-4.34) |
| Benishangul-Gumuz | 3.31 (2.99-3.81) | 3.94 (3.36-4.80) | 3.40 (3.07-3.91) | 3.59 (3.18-4.17) | 4.28 (3.61-5.36) | 3.68 (3.27-4.28) | 4.96 (4.41-5.72) | 5.74 (4.85-7.18) | 4.96 (4.41-5.72) |
| Dire Dawa | 2.69 (2.52-2.90) | 2.50 (2.30-2.80) | 2.35 (2.20-2.54) | 2.73 (2.55-2.97) | 2.54 (2.33-2.87) | 2.38 (2.21-2.60) | 3.24 (3.04-3.51) | 3.46 (3.18-3.89) | 3.24 (3.04-3.51) |
| Gambela | 2.13 (1.96-2.30) | 3.07 (2.46-4.03) | 2.31 (2.13-2.50) | 2.22 (2.08-2.38) | 3.21 (2.62-4.28) | 2.41 (2.26-2.59) | 2.89 (2.71-3.08) | 3.83 (3.11-5.17) | 2.89 (2.71-3.08) |
| Harari | 2.24 (2.10-2.43) | 3.49 (2.81-4.59) | 2.64 (2.47-2.87) | 2.41 (2.23-2.64) | 3.77 (3.06-5.04) | 2.84 (2.61-3.13) | 2.88 (2.67-3.16) | 3.79 (3.06-5.13) | 2.88 (2.67-3.16) |
| Oromiya | 2.39 (2.20-2.61) | 5.01 (3.80-7.04) | 3.49 (3.16-3.84) | 2.55 (2.36-2.80) | 5.38 (4.10-7.70) | 3.72 (3.34-4.14) | 3.42 (3.16-3.75) | 4.90 (3.77-6.99) | 3.42 (3.16-3.75) |
| SNNPR | 2.20 (2.03-2.46) | 4.06 (3.14-5.62) | 2.92 (2.66-3.26) | 2.36 (2.16-2.66) | 4.38 (3.41-6.20) | 3.14 (2.83-3.57) | 3.17 (2.91-3.55) | 4.40 (3.43-6.12) | 3.17 (2.91-3.55) |
| Somali | 3.04 (2.78-3.35) | 6.50 (4.73-9.39) | 4.25 (3.85-4.69) | 2.88 (2.68-3.11) | 6.20 (4.60-9.18) | 4.03 (3.65-4.42) | 4.16 (3.89-4.48) | 6.34 (4.73-9.47) | 4.16 (3.89-4.48) |
| Tigray | 2.30 (2.18-2.44) | 3.27 (2.91-3.80) | 2.91 (2.72-3.09) | 2.34 (2.20-2.49) | 3.33 (2.95-3.91) | 2.96 (2.74-3.17) | 3.16 (2.99-3.37) | 3.55 (3.19-4.15) | 3.16 (2.99-3.37) |

| (b) Measles | |  |  |  |  |  |  |  |  |
| --- | --- | --- | --- | --- | --- | --- | --- | --- | --- |
|  | 1. Deaths calculated independently from cases | | | 2. Deaths calculated with prognostic factors like risk factors and with treatment-seeking behavior applied | | | 3. Deaths calculated by applying under-5 mortality rates to cases | | |
|  | “Counterfactual” | Baseline | Maximized vaccination coverage | “Counterfactual” | Baseline | Maximized vaccination coverage | “Counterfactual” | Baseline | Maximized vaccination coverage |
| **Wealth quintile** |  |  |  |  |  |  |  |  |  |
| Poorest | 1.72 (1.55-1.97) | 2.33 (2.07-2.67) | 1.97 (1.77-2.26) | 1.94 (1.82-2.11) | 2.64 (2.42-2.89) | 2.23 (2.06-2.44) | 2.98 (2.77-3.22) | 3.52 (3.26-3.81) | 2.98 (2.77-3.22) |
| Poorer | 1.54 (1.40-1.77) | 1.94 (1.74-2.23) | 1.74 (1.57-2.00) | 1.69 (1.61-1.78) | 2.13 (2.00-2.28) | 1.91 (1.80-2.03) | 2.62 (2.48-2.76) | 2.92 (2.77-3.09) | 2.62 (2.48-2.76) |
| Middle | 1.37 (1.27-1.54) | 1.70 (1.57-1.93) | 1.52 (1.40-1.72) | 1.48 (1.42-1.53) | 1.84 (1.75-1.94) | 1.64 (1.57-1.72) | 2.05 (1.97-2.14) | 2.30 (2.20-2.42) | 2.05 (1.97-2.14) |
| Richer | 1.09 (1.00-1.20) | 1.15 (1.06-1.26) | 1.08 (1.00-1.19) | 1.07 (1.03-1.14) | 1.13 (1.09-1.21) | 1.06 (1.02-1.13) | 1.30 (1.25-1.38) | 1.38 (1.33-1.47) | 1.30 (1.25-1.38) |
| Richest | 1.00 (1.00-1.00) | 1.00 (1.00-1.00) | 1.00 (1.00-1.00) | 1.00 (1.00-1.00) | 1.00 (1.00-1.00) | 1.00 (1.00-1.00) | 1.00 (1.00-1.00) | 1.00 (1.00-1.00) | 1.00 (1.00-1.00) |
| **Region** |  |  |  |  |  |  |  |  |  |
| Addis Ababa | 1.00 (1.00-1.00) | 1.00 (1.00-1.00) | 1.00 (1.00-1.00) | 1.00 (1.00-1.00) | 1.00 (1.00-1.00) | 1.00 (1.00-1.00) | 1.00 (1.00-1.00) | 1.00 (1.00-1.00) | 1.00 (1.00-1.00) |
| Afar | 3.69 (3.04-4.78) | 6.31 (5.10-8.25) | 4.53 (3.69-5.91) | 4.89 (3.89-6.27) | 8.37 (6.50-10.77) | 6.01 (4.77-7.87) | 6.85 (5.43-8.76) | 9.54 (7.46-12.18) | 6.85 (5.43-8.76) |
| Amhara | 2.82 (2.34-3.66) | 4.62 (3.70-6.10) | 3.99 (3.22-5.27) | 3.65 (3.05-4.37) | 6.01 (4.89-7.38) | 5.20 (4.25-6.38) | 4.85 (4.02-5.76) | 5.61 (4.64-6.68) | 4.85 (4.02-5.76) |
| Benishangul-Gumuz | 3.23 (2.60-4.39) | 4.48 (3.56-6.14) | 4.08 (3.23-5.61) | 4.06 (3.24-5.19) | 5.64 (4.46-7.26) | 5.13 (4.06-6.61) | 5.55 (4.46-7.03) | 6.10 (4.89-7.70) | 5.55 (4.46-7.03) |
| Dire Dawa | 2.62 (2.18-3.37) | 3.18 (2.63-4.10) | 2.99 (2.47-3.84) | 3.17 (2.63-3.88) | 3.87 (3.18-4.73) | 3.63 (2.99-4.45) | 3.75 (3.08-4.52) | 3.99 (3.27-4.81) | 3.75 (3.08-4.52) |
| Gambela | 2.00 (1.77-2.33) | 2.96 (2.56-3.49) | 2.43 (2.13-2.85) | 2.40 (2.03-2.92) | 3.57 (2.97-4.39) | 2.93 (2.47-3.61) | 3.10 (2.63-3.75) | 3.78 (3.18-4.57) | 3.10 (2.63-3.75) |
| Harari | 2.35 (2.04-2.87) | 2.76 (2.39-3.37) | 2.35 (2.04-2.87) | 2.83 (2.43-3.31) | 3.32 (2.84-3.90) | 2.83 (2.43-3.31) | 3.35 (2.84-3.92) | 3.94 (3.33-4.61) | 3.35 (2.84-3.92) |
| Oromiya | 2.50 (2.14-3.14) | 4.64 (3.79-5.89) | 3.62 (2.98-4.61) | 3.06 (2.59-3.66) | 5.71 (4.63-7.06) | 4.46 (3.65-5.49) | 4.08 (3.42-4.85) | 5.22 (4.33-6.25) | 4.08 (3.42-4.85) |
| SNNPR | 2.31 (1.94-3.06) | 3.43 (2.81-4.57) | 2.86 (2.35-3.82) | 2.98 (2.51-3.59) | 4.45 (3.69-5.41) | 3.71 (3.09-4.56) | 3.98 (3.32-4.77) | 4.78 (3.96-5.76) | 3.98 (3.32-4.77) |
| Somali | 3.06 (2.60-3.74) | 5.49 (4.52-6.82) | 4.22 (3.49-5.22) | 3.80 (3.13-4.75) | 6.84 (5.47-8.64) | 5.27 (4.24-6.71) | 5.44 (4.43-6.74) | 7.07 (5.70-8.71) | 5.44 (4.43-6.74) |
| Tigray | 2.16 (1.85-2.67) | 3.15 (2.61-3.92) | 2.94 (2.44-3.68) | 2.64 (2.23-3.14) | 3.85 (3.13-4.66) | 3.61 (2.94-4.37) | 3.55 (2.99-4.16) | 3.79 (3.19-4.43) | 3.55 (2.99-4.16) |

| (c) Pneumococcal and Hib pneumonia | | |  |  |  |  |  |  |  |
| --- | --- | --- | --- | --- | --- | --- | --- | --- | --- |
|  | 1. Deaths calculated independently from cases | | | 2. Deaths calculated with prognostic factors like risk factors and with treatment-seeking behavior applied | | | 3. Deaths calculated by applying under-5 mortality rates to cases | | |
|  | “Counterfactual” | Baseline | Maximized vaccination coverage | “Counterfactual” | Baseline | Maximized vaccination coverage | “Counterfactual” | Baseline | Maximized vaccination coverage |
| **Wealth quintile** |  |  |  |  |  |  |  |  |  |
| Poorest | 1.64 (1.54-1.76) | 3.09 (2.51-3.95) | 2.35 (2.16-2.56) | 1.78 (1.66-1.94) | 3.38 (2.77-4.33) | 2.56 (2.33-2.83) | 2.74 (2.53-2.97) | 3.60 (2.96-4.60) | 2.74 (2.53-2.97) |
| Poorer | 1.43 (1.37-1.52) | 2.59 (2.19-3.19) | 2.07 (1.93-2.23) | 1.55 (1.47-1.66) | 2.81 (2.38-3.49) | 2.24 (2.05-2.45) | 2.40 (2.27-2.57) | 3.00 (2.55-3.67) | 2.40 (2.27-2.57) |
| Middle | 1.27 (1.24-1.31) | 2.13 (1.83-2.59) | 1.73 (1.64-1.81) | 1.33 (1.29-1.39) | 2.24 (1.92-2.72) | 1.82 (1.69-1.93) | 1.85 (1.79-1.92) | 2.27 (1.97-2.75) | 1.85 (1.79-1.92) |
| Richer | 1.01 (1.00-1.04) | 1.55 (1.40-1.76) | 1.37 (1.30-1.44) | 1.11 (1.08-1.15) | 1.69 (1.53-1.92) | 1.50 (1.41-1.57) | 1.34 (1.30-1.38) | 1.51 (1.39-1.70) | 1.34 (1.30-1.38) |
| Richest | 1.00 (1.00-1.01) | 1.00 (1.00-1.00) | 1.00 (1.00-1.00) | 1.00 (1.00-1.00) | 1.00 (1.00-1.00) | 1.00 (1.00-1.00) | 1.00 (NA-NA) | 1.00 (1.00-1.00) | 1.00 (1.00-1.00) |
| **Region** |  |  |  |  |  |  |  |  |  |
| Addis Ababa | 1.00 (1.00-1.00) | 1.00 (1.00-1.00) | 1.00 (1.00-1.00) | 1.00 (1.00-1.00) | 1.00 (1.00-1.00) | 1.00 (1.00-1.00) | 1.00 (1.00-1.00) | 1.00 (1.00-1.00) | 1.00 (1.00-1.00) |
| Afar | 3.63 (3.30-4.10) | 7.56 (5.14-11.62) | 4.40 (3.98-4.96) | 3.80 (3.42-4.30) | 7.98 (5.53-12.53) | 4.61 (4.12-5.21) | 5.39 (4.88-6.09) | 9.24 (6.35-14.74) | 5.39 (4.88-6.09) |
| Amhara | 2.70 (2.46-3.02) | 5.46 (4.25-7.43) | 4.07 (3.64-4.56) | 2.91 (2.64-3.27) | 5.89 (4.63-8.17) | 4.37 (3.87-5.00) | 3.88 (3.52-4.34) | 5.20 (4.12-7.10) | 3.88 (3.52-4.34) |
| Benishangul-Gumuz | 3.31 (2.99-3.81) | 3.94 (3.36-4.80) | 3.40 (3.07-3.91) | 3.59 (3.18-4.17) | 4.28 (3.61-5.36) | 3.68 (3.27-4.28) | 4.96 (4.41-5.72) | 5.74 (4.85-7.18) | 4.96 (4.41-5.72) |
| Dire Dawa | 2.69 (2.52-2.90) | 2.50 (2.30-2.80) | 2.35 (2.20-2.54) | 2.73 (2.55-2.97) | 2.54 (2.33-2.87) | 2.38 (2.21-2.60) | 3.24 (3.04-3.51) | 3.46 (3.18-3.89) | 3.24 (3.04-3.51) |
| Gambela | 2.13 (1.96-2.30) | 3.07 (2.46-4.03) | 2.31 (2.13-2.50) | 2.22 (2.08-2.38) | 3.21 (2.62-4.28) | 2.41 (2.26-2.59) | 2.89 (2.71-3.08) | 3.83 (3.11-5.17) | 2.89 (2.71-3.08) |
| Harari | 2.24 (2.10-2.43) | 3.49 (2.81-4.59) | 2.64 (2.47-2.87) | 2.41 (2.23-2.64) | 3.77 (3.06-5.04) | 2.84 (2.61-3.13) | 2.88 (2.67-3.16) | 3.79 (3.06-5.13) | 2.88 (2.67-3.16) |
| Oromiya | 2.39 (2.20-2.61) | 5.01 (3.80-7.04) | 3.49 (3.16-3.84) | 2.55 (2.36-2.80) | 5.38 (4.10-7.70) | 3.72 (3.34-4.14) | 3.42 (3.16-3.75) | 4.90 (3.77-6.99) | 3.42 (3.16-3.75) |
| SNNPR | 2.20 (2.03-2.46) | 4.06 (3.14-5.62) | 2.92 (2.66-3.26) | 2.36 (2.16-2.66) | 4.38 (3.41-6.20) | 3.14 (2.83-3.57) | 3.17 (2.91-3.55) | 4.40 (3.43-6.12) | 3.17 (2.91-3.55) |
| Somali | 3.04 (2.78-3.35) | 6.50 (4.73-9.39) | 4.25 (3.85-4.69) | 2.88 (2.68-3.11) | 6.20 (4.60-9.18) | 4.03 (3.65-4.42) | 4.16 (3.89-4.48) | 6.34 (4.73-9.47) | 4.16 (3.89-4.48) |
| Tigray | 2.30 (2.18-2.44) | 3.27 (2.91-3.80) | 2.91 (2.72-3.09) | 2.34 (2.20-2.49) | 3.33 (2.95-3.91) | 2.96 (2.74-3.17) | 3.16 (2.99-3.37) | 3.55 (3.19-4.15) | 3.16 (2.99-3.37) |

# References

1. Central Statistical Authority/Ethiopia, ORC Macro. Ethiopia Demographic and Health Survey 2000 [Internet]. Addis Ababa, Ethiopia: Central Statistical Authority/Ethiopia and ORC Macro; 2001. Available from: https://dhsprogram.com/publications/publication-fr118-dhs-final-reports.cfm

2. Central Statistical Authority/Ethiopia, ORC Macro. Ethiopia Demographic and Health Survey 2005 [Internet]. Addis Ababa, Ethiopia: Central Statistical Authority/Ethiopia and ORC Macro; 2006. Available from: https://dhsprogram.com/publications/publication-FR179-DHS-Final-Reports.cfm

3. Central Statistical Agency/CSA/Ethiopia, ICF International. Ethiopia Demographic and Health Survey 2011 [Internet]. Addis Ababa, Ethiopia: Central Statistical Agency and ICF International; 2012. Available from: http://dhsprogram.com/pubs/pdf/FR328/FR328.pdf

4. Central Statistical Agency/CSA/Ethiopia, ICF International. Ethiopia Demographic and Health Survey 2016 [Internet]. Addis Ababa, Ethiopia and Rockville, Maryland, USA: Central Statistical Agency and ICF International; 2016. Available from: http://dhsprogram.com/pubs/pdf/FR328/FR328.pdf

5. Black RE, Allen LH, Bhutta ZA, Caulfield LE, Onis M de, Ezzati M, et al. Maternal and child undernutrition: global and regional exposures and health consequences. The Lancet. 2008 Jan 19;371(9608):243–60.

6. Forouzanfar MH, Alexander L, Anderson HR, Bachman VF, Biryukov S, Brauer M, et al. Global, regional, and national comparative risk assessment of 79 behavioural, environmental and occupational, and metabolic risks or clusters of risks in 188 countries, 1990–2013: a systematic analysis for the Global Burden of Disease Study 2013. The Lancet. 2015 Dec 5;386(10010):2287–323.

7. Gebru T, Taha M, Kassahun W. Risk factors of diarrhoeal disease in under-five children among health extension model and non-model families in Sheko district rural community, Southwest Ethiopia: comparative cross-sectional study. BMC Public Health. 2014 Apr 23;14(1):395.

8. Global Burden of Disease Collaborative Network. Global Burden of Disease Study 2019 [Internet]. Seattle: Institute for Health Metrics and Evaluation (IHME); 2021. Available from: http://ghdx.healthdata.org/gbd-2019

9. Walker CLF, Rudan I, Liu L, Nair H, Theodoratou E, Bhutta ZA, et al. Global burden of childhood pneumonia and diarrhoea. The Lancet. 2013 Apr 20;381(9875):1405–16.

10. Sinmegn Mihrete T, Asres Alemie G, Shimeka Teferra A. Determinants of childhood diarrhea among underfive children in Benishangul Gumuz Regional State, North West Ethiopia. BMC Pediatr. 2014 Apr 14;14(1):102.

11. Li X, Mukandavire C, Cucunubá ZM, Londono SE, Abbas K, Clapham HE, et al. Estimating the health impact of vaccination against ten pathogens in 98 low-income and middle-income countries from 2000 to 2030: a modelling study. The Lancet. 2021 Jan 30;397(10272):398–408.

12. Munos MK, Walker CLF, Black RE. The effect of oral rehydration solution and recommended home fluids on diarrhoea mortality. Int J Epidemiol. 2010 Apr;39 Suppl 1:i75-87.

13. Vinodhini K, Shanmughapriya S, Das BC, Natarajaseenivasan K. Prevalence and risk factors of HPV infection among women from various provinces of the world. Arch Gynecol Obstet. 2012 Mar 1;285(3):771–7.

14. Bauer HM, Hildesheim A, Schiffman MH, Glass AG, Rush BB, Scott DR, et al. Determinants of Genital Human Papillomavirus Infection in Low-Risk Women in Portland, Oregon. Sex Transm Dis. 1993 Oct;20(5):274–8.

15. Belayneh T, Mitiku H, Weldegebreal F. Precancerous cervical lesion and associated factors among HIV-infected women on ART in Amhara Regional State, Ethiopia: A hospital-based cross-sectional study. Int J Health Sci. 2019 Jun;13(3):4–9.

16. Muñoz N, Franceschi S, Bosetti C, Moreno V, Herrero R, Smith JS, et al. Role of parity and human papillomavirus in cervical cancer: the IARC multicentric case-control study. The Lancet. 2002 Mar 30;359(9312):1093–101.

17. Centers for Disease Control and Prevention (CDC). STD Facts - Human papillomavirus (HPV) [Internet]. 2019 [cited 2020 Oct 6]. Available from: https://www.cdc.gov/std/hpv/stdfact-hpv.htm

18. Clarke B, Chetty R. Postmodern cancer: the role of human immunodeficiency virus in uterine cervical cancer. Mol Pathol. 2002 Feb;55(1):19–24.

19. Ferenczy A, Coutlée F, Franco E, Hankins C. Human papillomavirus and HIV coinfection and the risk of neoplasias of the lower genital tract: a review of recent developments. CMAJ Can Med Assoc J. 2003 Sep 2;169(5):431–4.

20. Palefsky JM, Minkoff H, Kalish LA, Levine A, Sacks HS, Garcia P, et al. Cervicovaginal Human Papillomavirus Infection in Human Immunodeficiency Virus-1 (HIV)-Positive and High-Risk HIV-Negative Women. JNCI J Natl Cancer Inst. 1999 Feb 3;91(3):226–36.

21. Wolfson LJ, Grais RF, Luquero FJ, Birmingham ME, Strebel PM. Estimates of measles case fatality ratios: a comprehensive review of community-based studies. Int J Epidemiol. 2009 Feb;38(1):192–205.

22. Zahidie A, Wasim S, Fatmi Z. Vaccine effectiveness and risk factors associated with measles among children presenting to the hospitals of Karachi, Pakistan. J Coll Physicians Surg. 2014 Dec;24(12):882–8.

23. Sudfeld CR, Navar AM, Halsey NA. Effectiveness of measles vaccination and vitamin A treatment. Int J Epidemiol. 2010 Apr;39 Suppl 1:i48-55.

24. Rudan I, Boschi-Pinto C, Biloglav Z, Mulholland K, Campbell H. Epidemiology and etiology of childhood pneumonia. Bull World Health Organ. 2008 May 1;86(5):408–16.

25. Bhat R, Manjunath N. Correlates of acute lower respiratory tract infections in children under 5 years of age in India. Int J Tuberc Lung Dis. 2013 Mar;17(3):418–22.

26. Lucero MG, Dulalia VE, Nillos LT, Williams G, Parreño RAN, Nohynek H, et al. Pneumococcal conjugate vaccines for preventing vaccine-type invasive pneumococcal disease and X-ray defined pneumonia in children less than two years of age. Cochrane Database Syst Rev. 2009 Oct 7;(4):CD004977.

27. Griffiths U, Clark A, Gessner B, Miners A, Sanderson C, Sedyaningsih E, et al. Dose-specific efficacy of Haemophilus influenzae type b conjugate vaccines: a systematic review and meta-analysis of controlled clinical trials. Epidemiol Infect. 2012 Aug;140(8):1343–55.

28. Theodoratou E, Johnson S, Jhass A, Madhi SA, Clark A, Boschi-Pinto C, et al. The effect of Haemophilus influenzae type b and pneumococcal conjugate vaccines on childhood pneumonia incidence, severe morbidity and mortality. Int J Epidemiol. 2010 Apr;39 Suppl 1:i172-185.
