# Supplementary material for: Modeling the relative risk of incidence and mortality of select vaccine-preventable diseases by wealth group and geographic region in Ethiopia
Source: PLOS Glob Public Health. 2022 Aug 31;2(8):e0000819. doi: 10.1371/journal.pgph.0000819 (PMC10004450; doi:10.1371/journal.pgph.0000819)
Supplement: Attachment — Submitted filename: Response_v3.docx [file pgph.0000819.s002.docx]

**To: *PLOS Global Public Health*
Subject: “Estimating the relative risk of incidence and mortality of select vaccine-preventable diseases by wealth group and geographic region in Ethiopia”**

June 28^th^, 2022

Dear Editors,

Thank you for the opportunity to submit a revision of our manuscript entitled “Estimating the relative risk of incidence and mortality of select vaccine-preventable diseases by wealth group and geographic region in Ethiopia”.

We would like to thank the reviewers and editors for their valuable and constructive comments on our work. In detail below, we give answers to the suggestions raised in the review.

Thank you for your consideration of our manuscript.

Sincerely,

Sarah Bolongaita, on behalf of the authors

# A. Journal Requirements

## 1. We ask that a manuscript source file is provided at Revision. Please upload your manuscript file as a .doc, .docx, .rtf or .tex. If you are providing a .tex file, please upload it under the item type ‘LaTeX Source File’ and leave your .pdf version as the item type ‘Manuscript’.

The manuscript has been uploaded as a .docx file.

## 2. Please provide separate figure files in .tif or .eps format only and remove any figures embedded in your manuscript file. Please ensure that all files are under our size limit of 20MB. If you are using LaTeX, you do not need to remove embedded figures. For more information about how to convert your figure files please see our guidelines: <https://journals.plos.org/globalpublichealth/s/figures>.

The figures have been removed from the manuscript and uploaded as separate .tif files.

## 3. We notice that your supplementary figures and tables are included in the manuscript file. Please remove them and upload them with the file type 'Supporting Information'. Please ensure that all Supporting Information files are included correctly and that each one has a legend listed in the manuscript after the references list.

The supplementary figures and tables have been removed from the manuscript and uploaded as supporting information files. A legend is included in the manuscript after the references list.

## 4. Please provide us with a direct link to the base layer of the map used in Figures 2 and 3, and ensure this location is also included in the figure legend. Please note that, because all PLOS articles are published under a CC BY license (creativecommons.org/licenses/by/4.0/), we cannot publish proprietary maps such as Google Maps, Mapquest or other copyrighted maps. If your map was obtained from a copyrighted source please amend the figure so that the base map used is from an openly available source. Please note that only the following CC BY licences are compatible with PLOS licence: CC BY 4.0, CC BY 2.0 and CC BY 3.0, meanwhile such licences as CC BY-ND 3.0 and others are not compatible due to additional restrictions. If you are unsure whether you can use a map or not, please do reach out and we will be able to help you. The following websites are good examples of where you can source open access or public domain maps: (i) U.S. Geological Survey (USGS) - All maps are in the public domain. (<http://www.usgs.gov>); (ii) PlaniGlobe - All maps are published under a Creative Commons license so please cite “PlaniGlobe, http://www.planiglobe.com, CC BY 2.0” in the image credit after the caption. (<http://www.planiglobe.com/?lang=enl>); (iii) Natural Earth - All maps are public domain. (<http://www.naturalearthdata.com/about/terms-of-use/>).

The base map used in Figures 2 and 3 is from GADM, which provides freely available data for academic use and other non-commercial use (<https://gadm.org/license.html>). The map data for Ethiopia can be found here: <https://gadm.org/download_country.html>.
In the manuscript, we added the sentence: “Base map data is available from GADM: <https://gadm.org/download_country.html>.” to the captions of Figures 2 and 3.

## 5. While revising your submission, please upload your figure files to the Preflight Analysis and Conversion Engine (PACE) digital diagnostic tool, <https://pacev2.apexcovantage.com/>. PACE helps ensure that figures meet PLOS requirements. To use PACE, you must first register as a user. Registration is free. Then, login and navigate to the UPLOAD tab, where you will find detailed instructions on how to use the tool. If you encounter any issues or have any questions when using PACE, please email PLOS at figures@plos.org. Please note that Supporting Information files do not need this step.

The figures were uploaded to PACE and converted to meet PLOS requirements prior to submission.

# B. Additional Editor Comments (if provided)

## 1. Please respond to the reviewer comments. In particular, please explain any differences between your papers' methodology and the Chang 2018 paper.

In the manuscript, we explain the differences between our methodology and the Chang et al. methodology from page 5 line 18 to page 6 line 3:

Chang and colleagues^10,16^ have proposed a first modeling approach to assess the distributional impact of vaccines on VPD incidence and mortality averted; however, their methodology did not yet account for the joint distribution of risk factors (i.e., the overlap between risk factors, that is accounting for individuals with multiple risk factors) and did not include variation by geographic region, neither did it examine the country of Ethiopia specifically. In this paper, we build on this preliminary work and significantly augment it in assessing the relative distribution of VPD incidence and mortality across socioeconomic strata and by geographic region in Ethiopia, taking into account the joint distribution of risk factors for these diseases.

and from page 7 line 11 to page 8 line 5:

Our approach substantially extends a previous methodology, which utilizes the population attributable fraction (PAF) and differences in the prevalence of risk and prognostic factors across wealth groups to estimate the distribution of VPD incidence and mortality across those wealth quintiles.^16^ In this paper, we augment this methodology by using a linear system of equations to estimate the joint distribution of risk and prognostic factors in population subgroups (i.e. wealth quintile and geographic region). To compute the joint distribution of risk and prognostic factors, we treat each possible combination of risk or prognostic factors as computationally distinct, thereby allowing us to account for individuals with multiple risk factors.

# C. Reviewers' Comments

## 1. Does this manuscript meet PLOS Global Public Health’s publication criteria? Is the manuscript technically sound, and do the data support the conclusions? The manuscript must describe methodologically and ethically rigorous research with conclusions that are appropriately drawn based on the data presented.

### Reviewer #1: Yes

### Reviewer #2: Partly

## 2. Has the statistical analysis been performed appropriately and rigorously?

### Reviewer #1: Yes

### Reviewer #2: No

## 1. Have the authors made all data underlying the findings in their manuscript fully available (please refer to the Data Availability Statement at the start of the manuscript PDF file)? The PLOS Data policy requires authors to make all data underlying the findings described in their manuscript fully available without restriction, with rare exception. The data should be provided as part of the manuscript or its supporting information, or deposited to a public repository. For example, in addition to summary statistics, the data points behind means, medians and variance measures should be available. If there are restrictions on publicly sharing data—e.g. participant privacy or use of data from a third party—those must be specified.

### Reviewer #1: Yes

### Reviewer #2: Yes

## 2. Is the manuscript presented in an intelligible fashion and written in standard English? PLOS Global Public Health does not copyedit accepted manuscripts, so the language in submitted articles must be clear, correct, and unambiguous. Any typographical or grammatical errors should be corrected at revision, so please note any specific errors here.

### Reviewer #1: Yes

### Reviewer #2: Yes

## 3. Review Comments to the Author: Please use the space provided to explain your answers to the questions above. You may also include additional comments for the author, including concerns about dual publication, research ethics, or publication ethics. (Please upload your review as an attachment if it exceeds 20,000 characters)

### Reviewer #1:

### In this manuscript, the authors employ an equity perspective and estimate the risk of incidence and mortality in selected vaccine-preventable diseases by household wealth groups and geographic regions in Ethiopia. In this modeling exercise, data from the Ethiopian Demographic and Health Survey were used to generate a simulated population, and the methodology was based on earlier published work. The authors present the modeling methodology and discuss its limitations.

### The empirical data used in the modeling emanate from the Ethiopian Demographic and Health Survey 2016, including data from almost a decade ago. The authors do not discuss the consequences for their estimates by the development during the years that have passed; first, increased coverage, followed by social insecurity, war, and pandemic.

We thank the reviewer for this thoughtful and beneficial review. Our analysis used data from the 2016 Ethiopian Demographic and Health Survey (DHS); the DHS represents the gold standard for empirical household survey data. While there was an Interim (or Mini) DHS conducted in Ethiopia in 2019, it did not contain all the necessary indicators for our model. We acknowledge that many events occurring in the interim period between 2016 and today, including the COVID-19 pandemic and civil unrest and armed conflict, could certainly impact some inputs in the model. However, these impacts would not affect the methodology of the paper and would not be likely to change some of the take-home messages of the analysis.
In the manuscript, we modified the sentence on page 21 lines 5-7 to include the phrase “somewhat outdated”:

First is the somewhat outdated data (from the DHS year 2016) and lacking evidence on risk and prognostic factors, especially for HPV, and the way in which these factors are estimated (i.e., compared to a null scenario, discussed above).

Later in the paragraph (page 21 lines 7-11), we added:

Much has happened since the 2016 Ethiopian DHS (e.g., the COVID-19 pandemic, armed conflict); however, the 2016 Ethiopian DHS remains the most recent source of comprehensive disaggregated (by wealth group and geographic region) empirical data on risk and prognostic factors relevant to this analysis.

### Inequities in coverage of health interventions depend on context, e.g., population and health system characteristics. This information is lacking in the manuscript. A minimum would be to briefly describe the health system and how vaccination services are organized in rural vs. urban areas and pastoralist vs. agrarian regions.

Thank you. We added this paragraph to the Introduction section beginning on page 4, line 17:

The country’s Expanded Programme on Immunization (EPI) is a core priority of the national Health Sector Transformation Plan (HSTP), wherein vaccines are provided routinely in health facilities, as well as in outreach and mobile health facilities and through vaccination campaigns.^12^ Ethiopia has developed several different strategies and plans to improve immunization coverage including utilization of the women (or health) development army (WDA), a women-centered community movement designed to improve the implementation capacity of the health system in rural areas (especially agrarian societies) through community engagement^12,13^, and comprehensive multiyear immunization plans (cMYP), which encompass all components of immunization services (e.g., service delivery, vaccine supply, quality and logistics, disease surveillance, advocacy, social mobilization and communication, and program management).^12,13^ However, effectively translating these strategies and plans into action has been challenging and community engagement has declined in recent years, with waning capacity and acceptability among WDA leaders and low acceptance by community members.^12,13^

### The authors conclude that their modeling shows that, given the inequities in using services, the health interventions (vaccination, treatment) worsen disparities. This result is not new information. It has been discussed in equity-focused publications from Ethiopia and Government policy documents for a relatively long time. My question is whether the authors may extract and discuss more policy-relevant conclusions?

We agree with the reviewer. We have now added these sentences to the Discussion section on page 20, lines 7-14:

This analysis highlights that poor and rural children in Ethiopia stand to benefit the most from more equitable vaccination programs because they would have the most to gain. Even pro-rich vaccination coverage, in which the richest quintiles have higher coverage rates (as observed in Ethiopia), likely result in greater health benefits in absolute terms (i.e., number of infections and deaths averted) given the more severe risk and prognostic profiles of the poor.^9^ Ethiopia’s vaccination program could become more pro-poor if coverage were more equal across wealth quintiles and the rural-urban divide, but might maintain relative health inequities if current pro-rich vaccine coverage rates remain.

### Reviewer #2:

### 1. The model simulates three scenarios – 1) no vaccination/ treatment seeking (counterfactual), 2) current vaccination and treatment seeking (baseline), and 3) maximized vaccination. It is counter intuitive why health outcomes are worsened in the baseline scenario. This was not the case for results reported by Chang et al. (2018). Since the current study was built on Chang et al.’s framework, please provide justification of this counter intuitive results.

We thank the reviewer for their thoughtful and thorough review. In our analysis, health outcomes in relative terms (i.e., the relative risk, not the outcomes in absolute terms) are worsened in the baseline scenario because of inequalities in vaccination coverage and treatment seeking behavior across populations belonging to different wealth quintiles and regions. The counterfactual scenario depicts inequalities related to underlying risk of vaccine-preventable diseases. Our results show that those who are poor and those who live in more rural regions have greater underlying risk of vaccine-preventable diseases due to the higher prevalence of disease risk factors. The baseline scenario depicts the relative impact of additional inequalities that stem from access to health services (i.e., vaccination coverage and treatment seeking behavior).

In simple terms, the baseline scenario shows an unequal situation made more unequal (compared with the counterfactual scenario). Health outcomes with underlying inequality (the counterfactual scenario based on risk factors for vaccine-preventable diseases) are worsened when access to health services is also considered, given that there are inequalities in access (the baseline scenario based on risk factors for vaccine-preventable diseases plus vaccination coverage and treatment seeking behavior). The inequalities observed in the counterfactual scenario and in access to health services compound to produce worsened health inequalities in relative terms in the baseline scenario.

We do not report on health outcomes in absolute terms; however, we agree that it would be unexpected to observe worse absolute health outcomes in the baseline scenario, when vaccination coverage and treatment seeking behavior are considered, compared to the counterfactual scenario. In the manuscript we state on page 19, lines 10-14:

In absolute terms, all population subgroups benefit from health interventions, however the distribution of health interventions across population subgroups may increase existing relative disparities. This is clearly not a reflection of the health interventions themselves, but is rather due to unequal access to care (often pro-rich gradients) through vaccination coverage and treatment-seeking behavior.

and on page 22, lines 7-10:

While vaccination undoubtedly improves health outcomes in absolute terms, the results of our modeling highlight the fact that unequal access to healthcare interventions can exacerbate pre-existing inequalities in the relative distribution of the risk and prognostic factors that give rise to VPD burden.

### 2. It is stated that “health interventions worsen the underlying inequalities observed in the counterfactual scenario”. What is the mechanism of this result?

See response to #1 above.

### 3. Does it mean abandoning vaccination programs will improve health outcomes? For example, Table S9 reports that at the poorest quintile, death from Rotavirus diarrhea is 1.64 in counterfactual and 2.35 in maximized coverage. Please explain this result. Also discuss what features of the model are driving this counter intuitive estimates.

Absolutely not. From our research we would certainly not recommend abandoning vaccination programs, especially since we only analyze and report *relative* health outcomes (i.e., the burden among the poor compared to the burden among the rich, not the averted *absolute* incidence or mortality). While current vaccination coverage (which is inequal across wealth quintiles and regions – i.e. pro-rich) can exacerbate *relative* inequalities in health outcomes, it certainly improves health outcomes in *absolute* terms. The population, regardless of their wealth quintile or region, benefits immensely from vaccination.

In the manuscript we state on page 19, lines 10-14:

In absolute terms, all population subgroups benefit from health interventions, however the distribution of health interventions across population subgroups may increase existing relative disparities. This is clearly not a reflection of the health interventions themselves, but is rather due to unequal access to care (often pro-rich gradients) through vaccination coverage and treatment-seeking behavior.

We did not examine impact on and reduction in absolute burden of vaccine-preventable diseases, which would be necessary to reliably report estimates of vaccine-preventable disease incidence and mortality (i.e. health outcomes in absolute terms); however, if we did, we would expect the baseline scenario to show improved health outcomes in absolute terms compared to the counterfactual scenario. These findings would not be in conflict with the findings of the research we conducted in this manuscript. Vaccination coverage, no matter how unequal, improves absolute health outcomes^1–7^; its impact on relative health outcomes, however, depends on the levels of inequality in coverage (which unfortunately tends to be pro-rich, as reported in the Ethiopian Demographic and Health Survey).
In the manuscript we state on page 20, lines 7-14:

This analysis highlights that poor and rural children in Ethiopia stand to benefit the most from more equitable vaccination programs because they would have the most to gain. Even pro-rich vaccination coverage, in which the richest quintiles have higher coverage rates (as observed in Ethiopia), likely result in greater health benefits in absolute terms (i.e., number of infections and deaths averted) given the more severe risk and prognostic profiles of the poor.^9^ Ethiopia’s vaccination program could become more pro-poor if coverage were more equal across wealth quintiles and the rural-urban divide, but might maintain relative health inequities if current pro-rich vaccine coverage rates remain.

### 4. Could you offer any theoretical explanation of the counter intuitive findings?

See responses to #1 and #3.

### 5. Could you report the aggregate incidence and mortality for each VPD in Table 3, in addition to results by quintiles and regions.

The purpose of this paper is on the examination of the relative distributional impact across wealth quintiles and geographic regions in Ethiopia. Therefore, unfortunately, we are not able to report on absolute aggregate incidence and mortality for each VPD.

### 6. What is the underlying mechanism of the model? Equitable vaccination coverage or higher vaccination coverage as a whole?

We are not quite sure what is meant by the “underlying mechanism of the model”, however the maximized vaccination coverage scenario represents both higher and more equitable vaccination coverage. The vaccination coverages of all population subgroups were set equal to the vaccination coverage of the population subgroup with the highest coverage (i.e., the vaccination coverages of quintiles one through four were set equal to the vaccination coverage of quintile five). This was done to show the impact of vaccination coverage that is equitable and feasible in practical terms (i.e., 100% vaccination coverage has not been achieved by any population subgroup and therefore may not be a practically relevant scenario to analyze; increasing coverage to the highest attained level reflects a scenario that may have more practical relevance).

### 7. Could you check another scenario with coverage rate be at the minimum (i.e., of the lowest quintile)? It is unclear from the analysis that whether more coverage is better or equal coverage is better. I think comparison of this new scenario with the baseline and maximized scenarios may provide some new perspectives.

Based on our modeling approach, we believe the results from a scenario in which the vaccination coverages of all population subgroups were set equal to the vaccination coverage of the population subgroup with the lowest coverage (i.e., the vaccination coverages of quintiles two through five were set equal to the vaccination coverage of quintile one) would mimic the results from the maximized vaccination coverage scenario. In both these scenarios, there is no relative difference in vaccination coverage between quintiles and regions. Regardless of whether vaccination coverage is increased to the maximum or reduced to the minimum, having equal vaccination coverage across population subgroups eliminates the contribution of vaccination coverage to the relative risk gradients. Furthermore, we see that a major objective of the Expanded Programme on Immunization is to both increase and equalize vaccination coverage to the highest possible levels (e.g. up to the vaccination coverage achieved by the richest quintile), which the maximized scenario materializes. As such, we feel that the “minimized scenario” proposed, which would decrease and equalize vaccination coverage to the lowest possible levels (e.g. down to the vaccination coverage achieved by the poorest quintile), while potentially interesting analytically, may bring confusing and mixed messages to the readers.

## 4. PLOS authors have the option to publish the peer review history of their article (what does this mean?). If published, this will include your full peer review and any attached files. Do you want your identity to be public for this peer review? If you choose “no”, your identity will remain anonymous but your review may still be made public. For information about this choice, including consent withdrawal, please see our Privacy Policy.

### Reviewer #1: Yes: Lars Ake Persson

### Reviewer #2: No
